# A novel role for OATP2A1/*SLCO2A1* in a murine model of colon cancer

**DOI:** 10.1038/s41598-017-16738-y

**Published:** 2017-11-29

**Authors:** Takeo Nakanishi, Yasuhiro Ohno, Rika Aotani, Shio Maruyama, Hiroaki Shimada, Shunsuke Kamo, Hiroko Oshima, Masanobu Oshima, John D. Schuetz, Ikumi Tamai

**Affiliations:** 1 Kanazawa University, Kanazawa, 920-1192 Japan; 2 Cancer Research Institute, Kanazawa University, Kanazawa, Japan; 3 Department of Pharmaceutical Sciences, St. Jude Children’s Research Hospital, Memphis, TN USA; 4 Present Address: Faculty of Pharmacy, Kindai University, Higashiosaka, Osaka, Japan

## Abstract

Prostaglandin E_2_ (PGE_2_) is associated with proliferation and angiogenesis in colorectal tumours. The role of prostaglandin transporter OATP2A1/*SLCO2A1* in colon cancer tumorogenesis is unknown. We evaluated mice of various *Slco2a1* genotypes in a murine model of colon cancer, the adenomatous polyposis (APC) mutant (*Apc* ^*∆716/*+^) model. Median lifespan was significantly extended from 19 weeks in *Slco2a1* ^+/+^/*Apc* ^*Δ716/*+^ mice to 25 weeks in *Slco2a1* ^−/−^/*Apc* ^*Δ716/*+^ mice. Survival was directly related to a reduction in the number of large polyps in the *Slco2a1* ^−/−^ */Apc* ^*∆716/*+^ compared to the *Slco2a1* ^+/+^/*Apc* ^*Δ716/*+^ or *Slco2a1* ^+/−^/*Apc* ^*Δ716/*+^mice. The large polyps from the *Slco2a1* ^−/−^ */Apc* ^*∆716/*+^ mice had significant reductions in microvascular density, consistent with the high expression of *Slco2a1* in the tumour-associated vascular endothelial cells. Chemical suppression of OATP2A1 function significantly reduced tube formation and wound-healing activity of PGE_2_ in human vascular endothelial cells (HUVECs) although the amount of extracellular PGE_2_ was not affected by an OATP2A1 inhibitor. Further an *in vivo* model of angiogenesis, showed a significant reduction of haemoglobin content (54.2%) in sponges implanted into *Slco2a1* ^−/−^, compared to wildtype mice. These studies indicate that OATP2A1 is likely to promote tumorogenesis by PGE_2_ uptake into the endothelial cells, suggesting that blockade of OATP2A1 is an additional pharmacologic strategy to improve colon cancer outcomes.

## Introduction

Colon cancer is one of the most common malignancies in humans and epidemiological studies have shown that ingesting aspirin, a nonsteroidal anti-inflammatory drug (NSAID), produces significant reductions in colorectal cancer death rate among individuals^1^. NSAIDs inhibit prostaglandin E_2_ (PGE_2_) production by COX^2,3^ and in animal models and humans inhibition of COX by NSAIDs suppresses colorectal tumour growth^4,5^. Genetic ablation of COX-1 or 2 in mice increases colon cancer survival rates, further supporting a role for suppressing PGE_2_ formation as a strategy to block colon cancer^6^. Multiple approaches have been described for disrupting the formation of PGE_2_ through COX inhibition^7,8^. However, COX-2, a rate-limiting enzyme of PGE_2_ synthesis, is upregulated in human colorectal tumours and their metastases^9,10^. This suggests that the dose of NSAID may be insufficient to inhibit the increased amounts of COX-2 in tumours and alternative strategies to disrupt the PGE_2_ pathway might be successful. For instance, the cell surface receptors for PGE_2_ are viable candidates because animals lacking the receptors EP1^11^ and EP2^12^ have increased survival rates in colon cancer models^12^. While PGE_2_ formation and its receptors contribute to colorectal cancer progression, it is unknown if the transporters which transport PGE_2_ have a role in colon cancer.

Extracellular PGE_2_ after binding to its cognate receptors activates a downstream signalling pathway that contributes to colon cancer progression by promoting the expression of genes involved in cell survival, tissue invasion and metastasis^8–10^. Generally anionic PGE_2_ does not readily cross biological membranes. The efficient release of PGE_2_ from cells requires a high affinity exporter, such as ABCC4^13^. PGE_2_ can be reimported and requires an uptake carrier^14–19^. The organic anion transporting polypeptide (OATP) 2A1 encoded by *SLCO2A1* (also known as PGT) is a high affinity uptake transporter for PGE_2_^17,18^. A previous study suggested that reduced OATP2A1 expression in colorectal carcinoma might enhance colorectal cancer^20^; however, there is no direct *in vivo* or *in vitro* evidence for OATP2A1 contributing to processes affecting colorectal tumour progression.

The present study evaluated if OATP2A1 expression impacted colorectal tumorigenesis in a murine model. To accomplish this, mice with or without *Slco2a1* were interbred with mice harbouring the APC adenomatous polyposis mutant (*Apc* ^*∆716/*+^) allele likely to mice prone gastrointestinal tumours^21^. Here, we present the first evidence that reduction in OATP2A1 levels or function has a beneficial role in promoting colon cancer survival by altering tumorigenesis.

## Results

### Impact of *Slco2a1* on colon cancer in a murine model (*Apc*^*Δ716/*^+)

**Figure 1:**
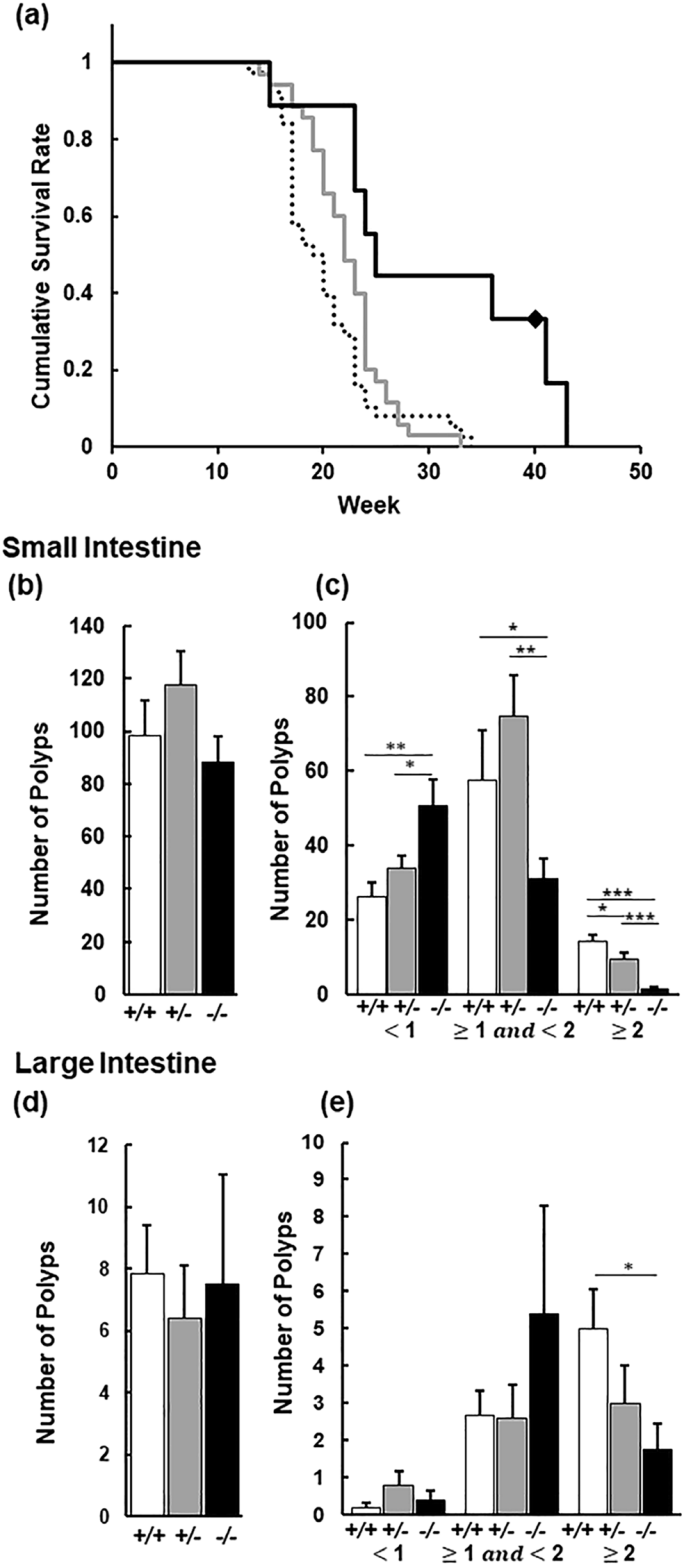
Impact of deletion of S*lco2a1* on survival and growth of intestinal polyps in *Apc* ^*Δ716/*+^ mice. (**a**) Survival curves of *Slco2a1* ^+*/*+^/*Apc* ^*∆716/*+^ (broken line, n = 38 including 23 males and 15 females), *Slco2a1* ^+/−^/*Apc* ^*Δ716/*+^ (grey line, n = 35 including 17 males and 18 females), and *Slco2a1* ^−/−^/*Apc* ^*Δ716/*+^ (solid line, n = 9 including 6 males and 3 females). Diamond shows censored data. Cumulative survival rate was analysed by Kaplan-Meier method for each cohort. (**b**,**d**) Number of overall total polyps in the small and large intestine in *Slco2a1* ^+*/*+^/*Apc* ^*∆716/*+^ (+/+, white), *Slco2a1* ^+/−^/*Apc* ^*Δ716/*+^ (+/−, grey), and *Slco2a1* ^−/−^/*Apc* ^*Δ716/*+^ (−/−, black). Number accounts for all polyps under the stereomicroscope. (**c**,**e**) Number of polyps are shown for three different size based on diameter in the small and large intestines. Each mouse was sacrificed at age of 13 weeks. Each bar represents the mean ± SEM. (n = 5–8). *, **, and *** indicate significant difference in polyp number of polyps by Student’s t-test with *p* < 0.05, <0.01, and <0.001, respectively.

Mice lacking *Slco2a1*were intercrossed with mice harbouring a truncated form of the adenomatous polyposis coli gene (*APC*/*Apc*) (*Apc* ^*Δ716/*+^) which predisposes mice to lethal gastrointestinal tumours^21^. The absence of *Slco2a1* promoted survival as the median lifespan of 19 weeks in *Slco2a1* ^+*/*+^/*Apc* ^*Δ716/*+^ mice (n = 38) was extended to 22 and 25 weeks in *Slco2a1* ^+/−^/*Apc* ^*Δ716/*+^ (n = 35) and *Slco2a1* ^−/−^/*Apc* ^*Δ716/*+^ mice (n = 9), respectively (p = 0.008 and 0.005) (Fig. 1a). These findings suggest that lower Oatp2a1 levels are protective and provide a survival advantage, whereas higher levels are not.

We next assessed if polyp formation by *Apc* ^*Δ716/*+^ was altered by the amount of Oatp2a1. Mice were examined at 13 weeks for the polyp size and number in the small and large intestines of the *Apc* ^*Δ716/*+^ mutant mice that had been intercrossed with mice having either a single or no *Slco2a1* allele. The total number of polyps in the small intestine of *Slco2a1* ^+*/*+^/*Apc* ^*Δ716/*+^, *Slco2a1* ^+/−^/*Apc* ^*Δ716/*+^, and *Slco2a1* ^−/−^/*Apc* ^*Δ716/*+^ mice were 98.2 ± 13.4, 117.6 ± 12.8, and 88.0 ± 9.9, respectively. Although there was no significant difference in total polyp number observed between the *Slco2a1* genotypes (Fig. 1b), the size of the polyps was affected by the *Slco2a1* genotype. Notably, polyps less than 1 mm in diameter were more frequent in *Slco2a1* ^−/−^/*Apc* ^*Δ716/*+^ mice and accounted for more than 50% of the total polyps. In contrast, larger polyps (between 1-to-2 mm as well as >2 mm) were significantly less frequent in the *Slco2a1* ^−/−^/*Apc* ^*Δ716/*+^ mice than those in *Slco2a1* ^+*/*+^/*Apc* ^*Δ716/*+^ and *Slco2a1* ^+/−^/*Apc* ^*Δ716/*+^ mice. Large polyps (>2 mm) in *Slco2a1* ^−/−^/*Apc* ^*Δ716/*+^ mice accounted for only 1.3 ± 0.59% of total polyps vs. over 10% of the polyps in the *Slco2a1* ^+*/*+^/*Apc* ^*∆716/*+^ mice (Fig. 1c). There was no significant difference in the total polyps in the large intestine between the three *Slco2a1* genotypes (Fig. 1d). However, larger polyps were significantly less frequent in the *Slco2a1* ^−/−^/*Apc* ^*Δ716/*+^ mice (Fig. 1e). The EP4 receptor appears to contribute to colon carcinogenesis^22,23^. Expression of the EP4 receptor in the polyps from the small intestine revealed comparable amounts in *Slco2a1* ^+/+^/*Apc* ^Δ716/+^ and *Slco2a1* ^−/−^/*Apc* ^Δ716/+^ mice (Supplementary Figure S1).

### *Slco2a1* deficiency affects microvascular density (MVD) in the small intestine

**Figure 2:**
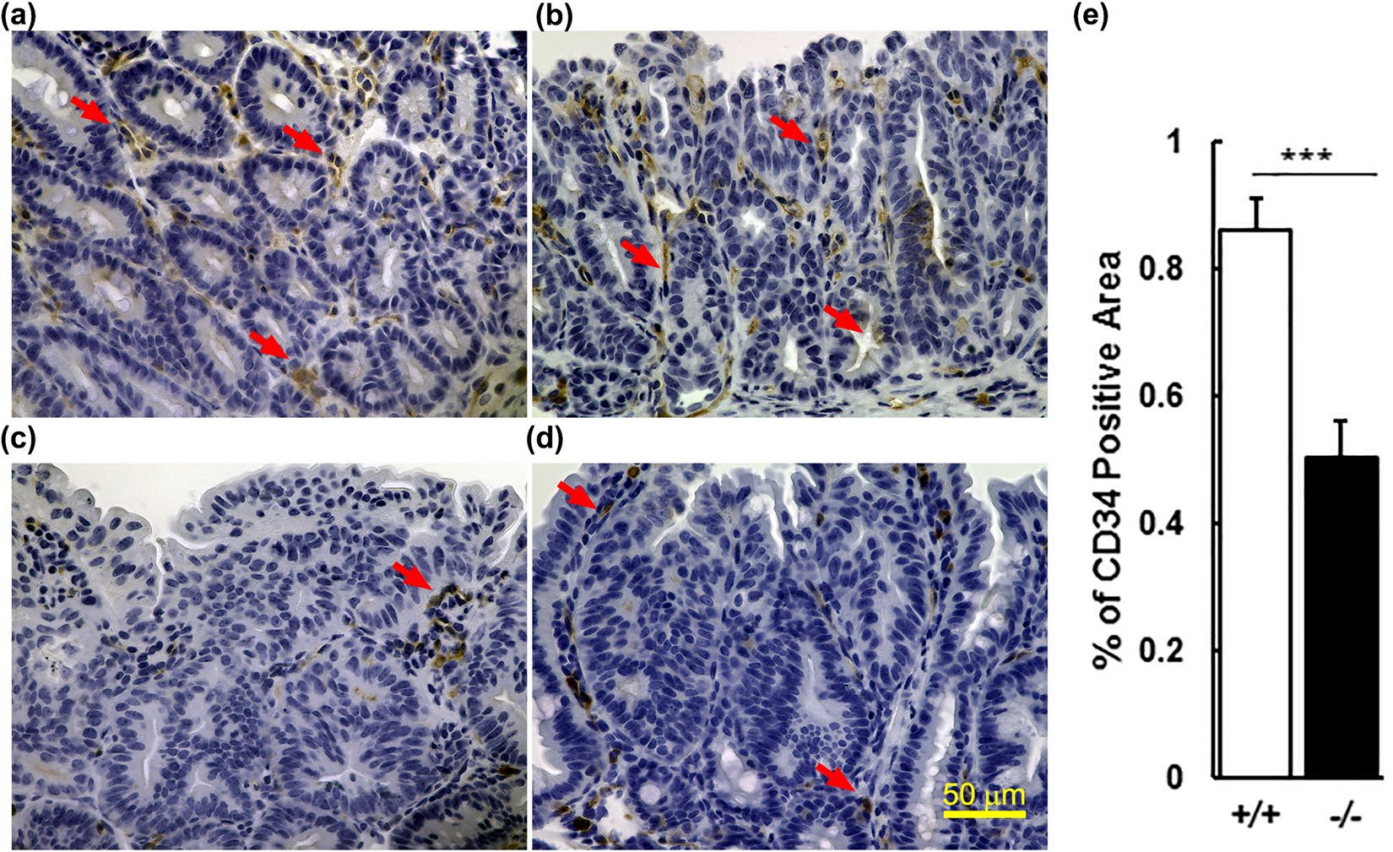
Effect of *Slco2a1* on MVD in intestinal polyps. CD34 expression in paraffin section (4 μm) of polyps in small intestines prepared from two different *Slco2a1* ^+*/*+^/*Apc* ^*∆716/*+^ (+/+) (**a,b**) and *Slco2a1* ^−/−^/*Apc* ^*Δ716/*+^ (−/−) (**c**,**d**). Pictures were taken under BZ-9000 with a magnification (× 40). The section was incubated with anti-CD34 antibody and stained brown by immunoenzymatic reaction with DAB. Typical DAB stain is indicated by red arrows. Nuclei were counter-stained blue with haematoxylin. Indicated arrows show immunoreactivity for anti-CD34 antibody. Experiments were repeated at least three times, and most representative pictures are shown. (**e**) CD34 positive area (%) in the region of determined polyps. The region of interest was quantitatively determined by Image J and bar graph represents the mean values of 48 individual section from 4 mice (12 sections from each). *** indicates significant difference by Student’s t-test with *p* < 0.001.

The size of colon cancer polyps has been related to vasculature and angiogenesis^24^. MVD is often an indicator of angiogenic capability of endothelial cells^25^. To quantify the MVD in polyps from the small intestine, an antibody against the endothelial marker CD34 was used. The extent of the CD34-positive area was compared in intestinal polyps between the *Slco2a1* ^+*/*+^/*Apc* ^*∆716/*+^ (Fig. 2a and b) and *Slco2a1* ^−/−^/*Apc* ^*Δ716/*+^ (Fig. 2c and d) mice. The CD34 immunoreactivity was more frequently detected in the polyps taken from *Slco2a1* ^+*/*+^ */Apc* ^*Δ716/*+^ mice. The proportion of CD34-positive area within the polyps of *Slco2a1* ^−/−^/*Apc* ^*Δ716/*+^ mice was reduced to 58.7%, and significantly lower (p = 10^−5^) than that in *Slco2a1* ^+*/*+^ */Apc* ^*Δ716/*+^ mice (Fig. 2e). This result suggests that angiogenesis is suppressed in the absence of *Slco2a1*.

### Immunolocalization of Oatp2a1 in intestinal polyps

**Figure 3:**
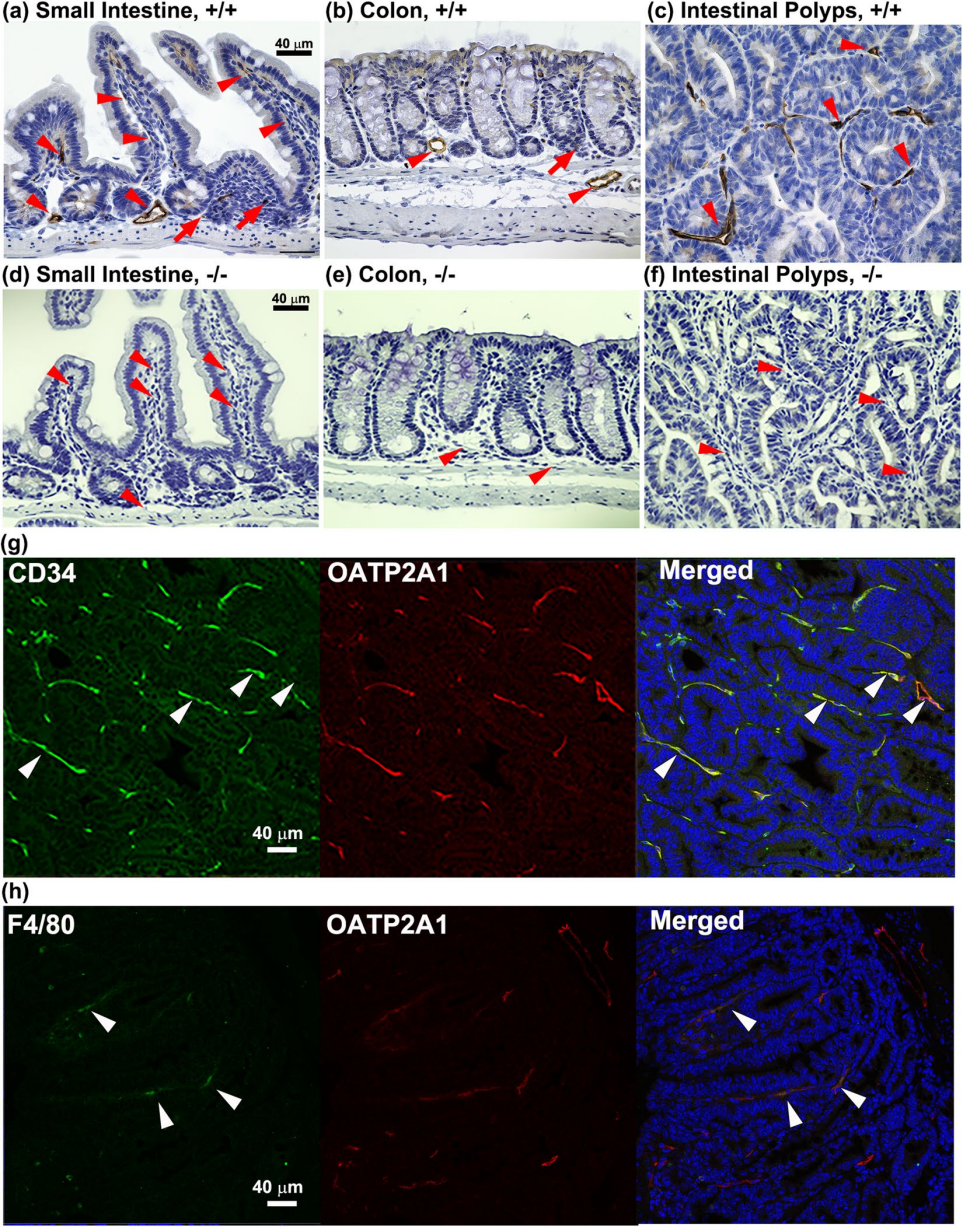
Immunohistochemical localization of Oatp2a1 in normal gut and intestinal polyps. Paraffin section (4 μm) of mouse small intestine (**a**,**d**), colon (**b**,**e**), and intestinal polyps (**c**,**f**) of *Slco2a1* ^+*/*+^/*Apc* ^*∆716/*+^ (+/+, **a**–**c**) and *Slco2a1* ^−/−^/*Apc* ^*Δ716/*+^ (−/−, **d**–**f**) was incubated with anti-Oatp2a1 antibody and stained brown by immunoenzymatic reaction with DAB. Nuclei were counter-stained blue with haematoxylin. Red arrowhead and arrow indicate endothelial and stromal cells, respectively. Co-localization of CD34 (**g**, green) or F4/80 (**h**, green) with Oatp2a1 (red) was detected in polyps of small intestines of *Slco2a1* ^+*/*+^/*Apc* ^*∆716/*+^. Nuclei were counterstained with Hoechst33342 (blue). Indicated white arrowheads show typical positive fluorescence for each reaction. Experiments were repeated at least three times and representative images are shown.

Immunohistochemical analysis was performed to determine Oatp2a1 expression in normal small intestine and colon compared to polyp in the small intestine of *Slco2a1* ^+*/*+^ */Apc* ^*Δ716/*+^ mice (Fig. 3a–c). Oatp2a1 protein was prominent in the blood vessel endothelia (as indicated by a red arrowhead), but also in some cells with a round morphology (by red arrows) in the stromal tissues of the normal small intestine (Fig. 3a) and colon (Fig. 3b) of *Slco2a1* ^+*/*+^/*Apc* ^*∆716/*+^ mice. Notably, the Oatp2a1 immunoreactivity appears much more intense in the stromal tissues of intestinal polyps of *Slco2a1* ^+*/*+^ */Apc* ^*Δ716/*+^ mice (Fig. 3c), compared to the much weaker immunoreactivity in the normal small intestine and colon. As expected no Oatp2a1 immunoreactivity was detected in the corresponding region of *Slco2a1* ^−/−^ */Apc* ^*Δ716/*+^ mice (Fig. 3d–f). To confirm Oatp2a1-expression in endothelial cells (versus macrophage), intestinal polyp samples were prepared from *Slco2a1* ^+*/*+^ */Apc* ^*Δ716/*+^ mice and co-labelled with anti-CD34 (Fig. 3g) or anti-F4/80 (a marker of activated macrophage) (Fig. 3h) and anti-Oatp2a1 antibody. Red fluorescence for Oatp2a1 mostly co-localized with the green fluorescence for anti-CD34 (Fig. 3g, merged). Red fluorescence only partly co-localized with green for anti-F4/80 (Fig. 3h, merged), indicating Oatp2a1 is primarily expressed in vasculature endothelial cells.

### Role of OATP2A1 on angiogenic capability of HUVECs

**Figure 4:**
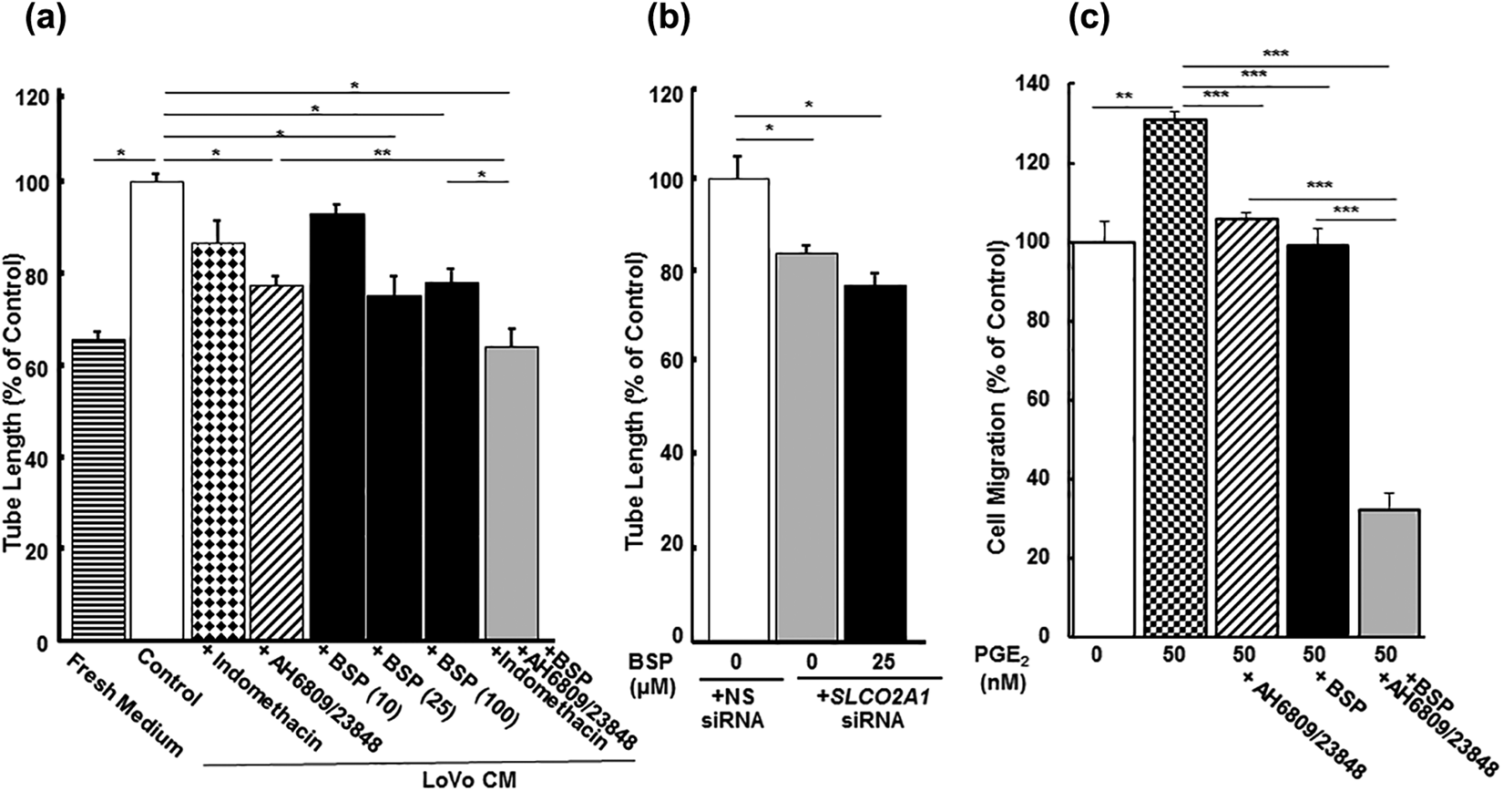
Angiogenic capability of HUVECs is affected by OATP2A1. Tube formation by HUVECs was measured in the CM from human colorectal cancer LoVo cells, which were incubated in Ham’s F-12 for 24 h (**a**). HUVECs were incubated with fresh medium (stripe) or the CM in the absence (as control, open) or the presence of indomethacin (10 µM, diamond), mixture of EP antagonists (AH6809; 10 µM, AH23848; 25 µM, hatched), BSP (10, 25, or 100 µM, closed), or all agents (grey) on Matrigel® for 4 h. Tube formation assay was observed in HUVECs knocked-down for *SLCO2A1* (**b**). Wound healing assay of HUVECs untreated (as control, open) or treated with PGE_2_ (50 nM, check) in the absence or presence of mixture of EP antagonists (AH6809; 10 µM and AH23848; 25 µM, hatched), BSP (25 µM, closed), or the both (grey) for 10 h (**c**). Experiments were repeated at least three times with triplicate, and each point represents results with the mean ± SEM. (n = 3). *, ** and *** indicate significant difference in tube length from by Student’s t-test with *p* < 0.05, <0.01 and <0.001, respectively.

Both the formation of tube-like structures and endothelial cell migration are hallmark features of angiogenesis^26^. Transformed epithelial cells release many soluble factors, including PGE_2_^27^. To assess the role of soluble factors and OATP2A1, the “tube formation” assay in HUVECs was used. Our initial experiments used conditioned medium (CM) from the colon cancer, LoVo cell line. The PGE_2_ concentration in the CM from LoVo cells was approximately 6-fold higher than fresh medium (23.2 ± 3.13 vs 4.0 ± 1.23 pM), and HUVECs treated with CM significantly increased the total tube length by 52.7% (Fig. 4a). Tube formation observed in CM was reduced by the presence of either the COX inhibitor, indomethacin, or the EP antagonists, AH6809 and AH23848. BSP, an OATP2A1 inhibitor, suppressed tube formation modestly at 10 μM and and maximally at 100 μM. The reduced tube formation displayed by indomethacin and EP antagonists was further decreased by the addition of BSP. Moreover, HUVEC tube formation was significantly reduced by silencing *SLCO2A1* and as expected it was not further suppressed by the presence of BSP (Fig. 4b). The *SLCO2A1* mRNA was decreased to a level that was 12.8% of control cultures transfected with the NS siRNA HUVECs (n = 6, p = 0.0001). Because solutes other than PGE_2_ might produce the effects observed by the CM, the impact of PGE_2_ on HUVECs was assessed by wound healing assay in FBS-free EBM™ supplemented with EGM™-2. HUVEC migration significantly increased by 31% in the presence of PGE_2_ (Fig. 4c). Migratory activity of HUVECs treated with PGE_2_ was reduced significantly in the presence of the AH6809 and AH23848, or BSP, and further declined in the presence of the both antagonists and BSP as observed in the tube formation assays undertaken in LoVo CM.

**Figure 5:**
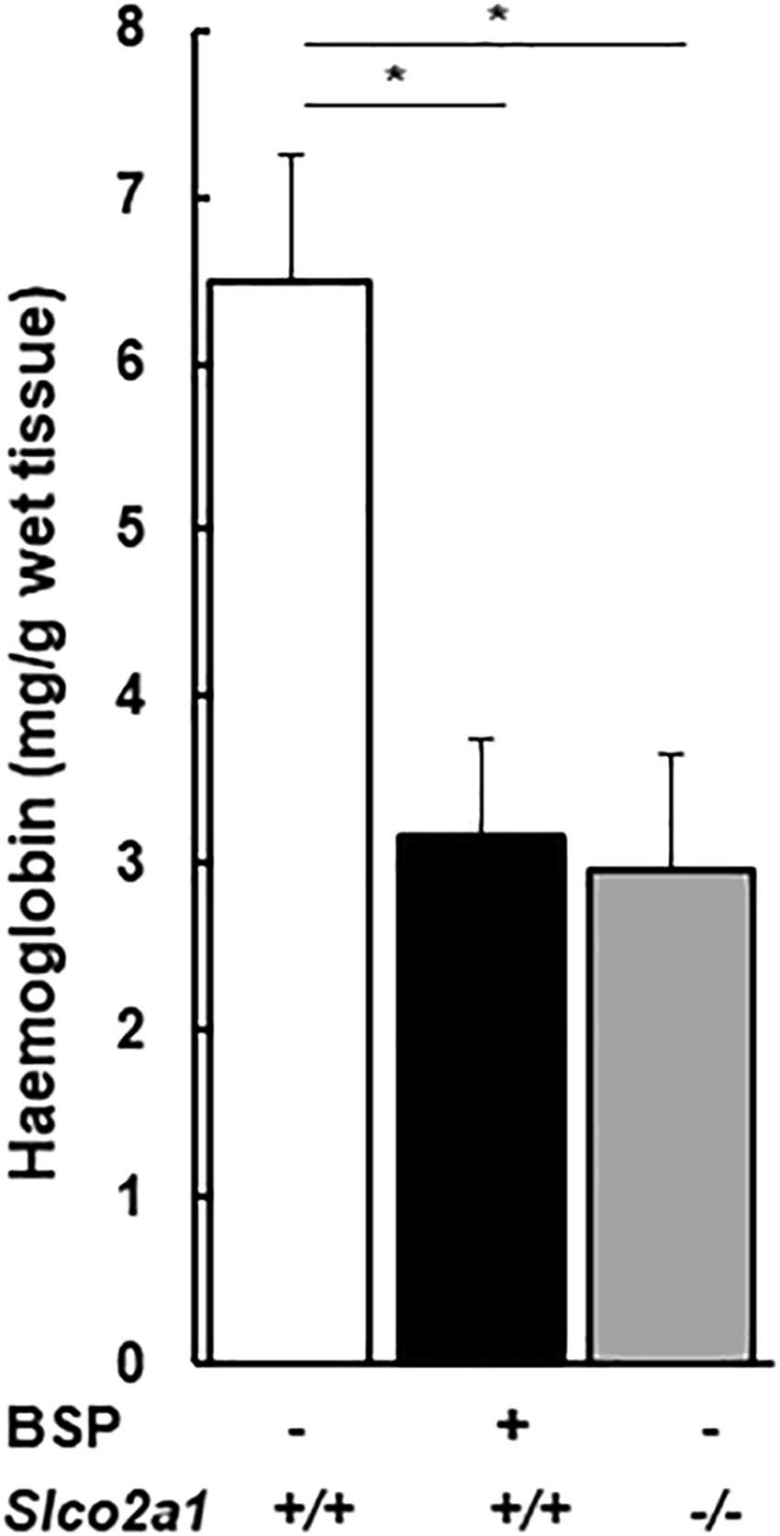
Impact of Oatp2a1 on *in vivo* angiogenic capability of endothelial cells. Haemoglobin was measured in implanted sponges in *Slco2a1* wildtype (+/+, white bar) or *Slco2a1* ^−/−^ (−/−, grey) mice (with mixed genetic background of BL/6 and SV129). BSP in physiological saline (5 nmol/100 μL/day/mouse) was consecutively injected into the sponge for 9 days (black), otherwise mice were injected with sterilized physiological saline. On Day 10, haemoglobin content was measured and normalized with wet weight of sponge granular tissues. Each bar represent of the mean value of 3–8 mice with SEM. * indicates significant difference in tube length from by Student’s t-test with *p* < 0.05, and <0.01, respectively.

To determine the impact of OATP2A1 on angiogenesis *in vivo*, we used the sponge subcutaneous implantation model^28^ and measured haemoglobin content because it correlates with angiogenesis in this model^29^. We compared haemoglobin content of the sponge granular tissue in *Slco2a1* ^+/+^ and *Slco2a1* ^−/−^ mice that were implanted with a sponge that had been injected with either vehicle or BSP. Haemoglobin content was reduced by 54.2% in *Slco2a1*-deficient mice, compared to *Slco2a1* ^+/+^ mice. Further, sponge treatment with BSP produced a comparable reduction in haemoglobin content. Therefore, these results strongly indicate that OATP2A1 function is required to promote angiogenesis (Fig. 5).

### Expression of functional OATP2A1 in HUVECs

**Figure 6:**
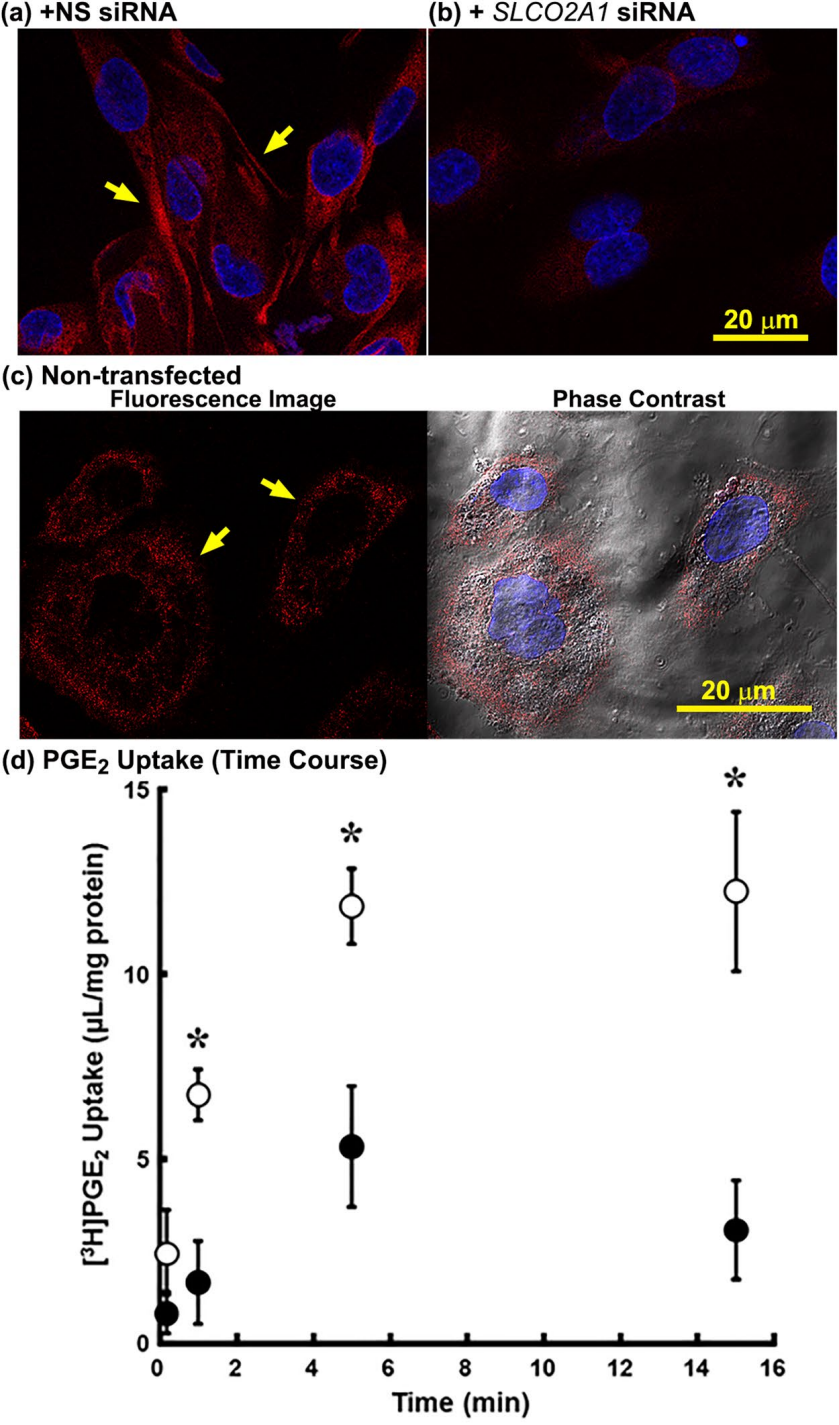
OATP2A1 function affects PGE_2_ accumulation in HUVECs. Fluorescence immunocytochemistry was performed with anti-OATP2A1 antibody in HUVECs transfected with NS siRNA (**a**) or *SLCO2A1* siRNA (**b**), and none (**c**), followed by counterstaining with Hoechst 33342 for nuclei (blue). Fluorescence was merged with confocal phase contrast (**c**, phase contrast). Yellow arrow indicates fluorescence on the plasma membranes. Experiments were repeated at least three times, and representative image is shown. Uptake of [^3^H]PGE_2_ (1.5 nM) by HUVECs (**d**). Cellular uptake was observed in the absence (as control, open circle) or presence of BSP (25 µM, closed circle) over 15 min at 37 °C and pH 7.4. Each point represents the mean ± SEM. (n = 3). * indicates significant difference in uptake from control value by Student’s t-test with *p* < 0.05.

Expression of OATP2A1 was investigated in HUVECs by means of immunocytochemistry. The red fluorescence revealed by the anti-OATP2A1 antibody indicated OATP2A1 is mostly at the plasma membranes of HUVECs transfected with NS siRNA (Fig. 6a). The specificity of the anti-OATP2A1 is shown by the strong reduction in the immunodetectable OATP2A1 in the *SLCO2A1* siRNA (Fig. 6b). The merged optical/fluorescence images of HUVECs show that OATP2A1 primarily localizes to the plasma membrane and/or submembranous structure (Fig. 6c). Accordingly, the [^3^H]PGE_2_ uptake by HUVECs and its intracellular accumulation at steady state were significantly reduced in the presence of BSP (Fig. 6d), consistent with the role of OATP2A1 in determining uptake and intracellular accumulation of PGE_2_.

### Effect of PGE_2_ taken up by HUVECs on their migratory activity

**Figure 7:**
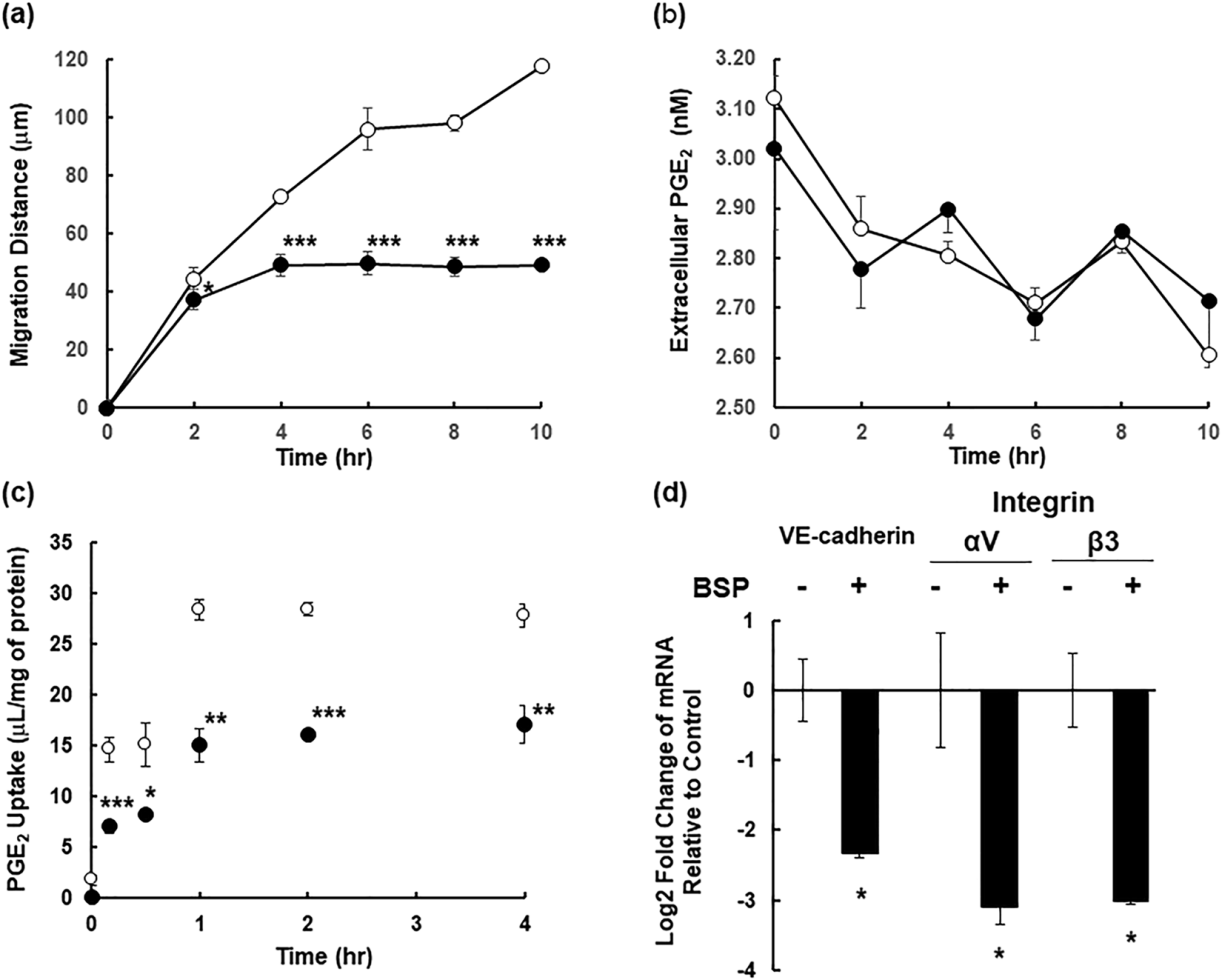
Effect of exogenous PGE_2_ on Migratory Capability of HUVECs. (**a**) Migratory activity of HUVECs pre-treated with indomethacin (100 μM) for 16 hrs. Migration distance of HUVECs was determined in EGM-2 containing PGE_2_ (3 nM) and indomethacin (100 μM) in the absence (Control, open circles) or the presence of BSP (25 μM, closed circles). Each point represents the mean ± SEM. (n = 4). (**b**) Extracellular PGE_2_ concentration was monitored for 10 hrs under the same condition. (**c**) Cellular uptake of [^3^H]PGE_2_ (10 nM) by HUVECs. HUVECs were pre-incubated with EGM-2 containing indomethacin (100 μM) for 16 hrs before experiment. Uptake was measured in the absence (Control, open circles) or presence of BSP (25 µM) (closed circles) at 37 °C and pH 7.4. Each point represents the mean ± SEM. (n = 3). (**d**) mRNA expression of cell adhesion-related genes (e.g. VE-cadherin, integrin αV and integrin β3) was evaluated by quantitative RT-PCR. HUVECs were pre-incubated with EGM-2 containing indomethacin (100 μM) for 16 hrs before experiment. Then, HUVECs were incubated with EGM-2 containing indomethacin in the absence (Control) or presence of BSP (25 μM, closed columns) for 10 hrs. Each bar represents the mean ± SEM. (n = 3). * indicates the significant different expression from Control by Students t-test (p < 0.05).

Finally, to determine whether PGE_2_ taken up by cells is involved in angiogenesis, HUVECs were treated with indomethacin for 16 hrs, and then the impact of BSP on their migratory activity was assessed for 10 hrs in the presence of PGE_2_. For control cells, the migration distance increased in a time-dependent manner. In contrast BSP-treated cells showed reduced migration at every time point (Fig. 7a). Unexpectedly, there was no significant difference in extracellular PGE_2_ between untreated and BSP-treated HUVECs (Fig. 7b). Consistent with the migration assay, under the same condition, intracellular accumulation of [^3^H]PGE_2_ in HUVECs was significantly reduced by BSP, and reached a plateau in 1 hr (Fig. 7c). Moreover, the effect of BSP on mRNA expression of adhesion molecules, which play critical role in migration, in HUVECs was determined by quantitative RT-PCR (Fig. 7d). mRNA expression of VE-cadherin, integrin αV and β3 was significantly decreased in HUVECs treated with BSP. These data suggest that PGE_2_ taken up by cells plays a role in migration of endothelial cells independently cell surface EP receptor.

## Discussion

Previous studies have established that PGE_2_, a product derived from arachidonic acid via COX, facilitates colorectal tumour progression and that either disruption of its synthesis or the blockade of prostanoid E receptors delays disease progression^5,6,8–10^, strongly suggesting that interfering with this pathway improves colon cancer survival. However, the spectrum of potential contributors to the PGE_2_ pathway is incomplete. One candidate is OATP2A1, a transporter that has been suggested^20^, but not demonstrated, to affect PGE_2_ uptake in colon cancer cells^30,31^. However, to date, there is no definitive studies that have shown that OATP2A1 affects the genesis of colon cancer. The present study demonstrated that genetic ablation of *Slco2a1* strongly enhanced survival and this was associated with a marked suppression in the number of large colorectal cancer polyps.

The expression of the EP4 receptor is associated with colon cancer tumorogenesis^22,23^. We hypothesized that changes in the expression of the EP4 receptor accounts for the increased survival in the *Slco2a1-null* mice. Our immunohistochemical analysis indicated that EP4 receptor expression was similar among *Slco2a1-null* and wildtype mice (Supplementary Figure S1), suggesting that it does not account for the reduced tumorogenesis among *Slco2a1-null* mice. It is conceivable that PGE_2_ is elevated in the extracellular space of *Slco2a1-null* tumours, however, at this time we are not able to reliably measure the PGE_2_ in the extracellular space of tumours.

How does absence of *Slco2a1* affect colon cancer tumorogenesis? Our studies support a mechanism whereby OATP2A1 expression in the endothelial vasculature of the tumour, is required for maximal tumorigenesis in the APC mutant (*Apc* ^*∆716/*+^) mouse model. While the number of small polyps was not impacted by *Slco2a1* absence, the formation of large malignant polyps was markedly reduced by the loss of Oatp2a1 (Fig. 1). Notably, the endothelial marker CD34 (a marker of endothelial vasculature), was significantly lower in the stromal tissue of intestinal polyps from *Slco2a1*-deficient *Apc* ^*Δ716/*+^ mice (Fig. 2). This is consistent with findings showing increased CD34 levels in colorectal tumours^32,33^ are a significant and independent poor prognostic factor in colon cancer^34^, and would be in accord with the finding that angiogenesis correlated with the size of malignant adenoma polyps^8,9^. Our *in vivo* studies showed Oatp2a1 was expressed in the tumour’s endothelial vasculature, which agrees with the previous study in human small intestines^35^. We speculate that OATP2A1 plays a role in neovascularization because vascular stresses are known factors to induce OATP2A1 expression^36^. However, it is currently unclear exactly how OATP2A1 expression in the vasculature of endothelial cells contributes to tumour angiogenesis. The present findings might be unique to the tumour micro-environment because Syeda *et al*. reported that pharmacological inhibition of Oatp2a1 in a diabetic mouse model promoted angiogenesis^30,37^. Nonetheless, the reduced microvascular density in *Slco2a1*-deficient polyps coupled with OATP2A1 affecting angiogenesis, as shown both *in vitro* tube formation and wound healing assays in HUVECs (Fig. 4) and *in vivo* sponge subcutaneous implantation model (Fig. 5), suggests that OATP2A1 contributes to tumour angiogenesis. Moreover, under PGE_2_-depeleted conditions, migration of HUVECs were associated with intracellular PGE_2_ levels rather than extracellular PGE_2_, and cell adhesion-related gene expressions were down-regulated (Fig. 7). Suppression of angiogenesis is related to decreased OATP2A1-mediated uptake both *in vitro* and *in vivo* and supports the idea that OATP2A1-mediated transport of PGE_2_ contributes to angiogenesis independent of EP receptors.

**Figure 8:**
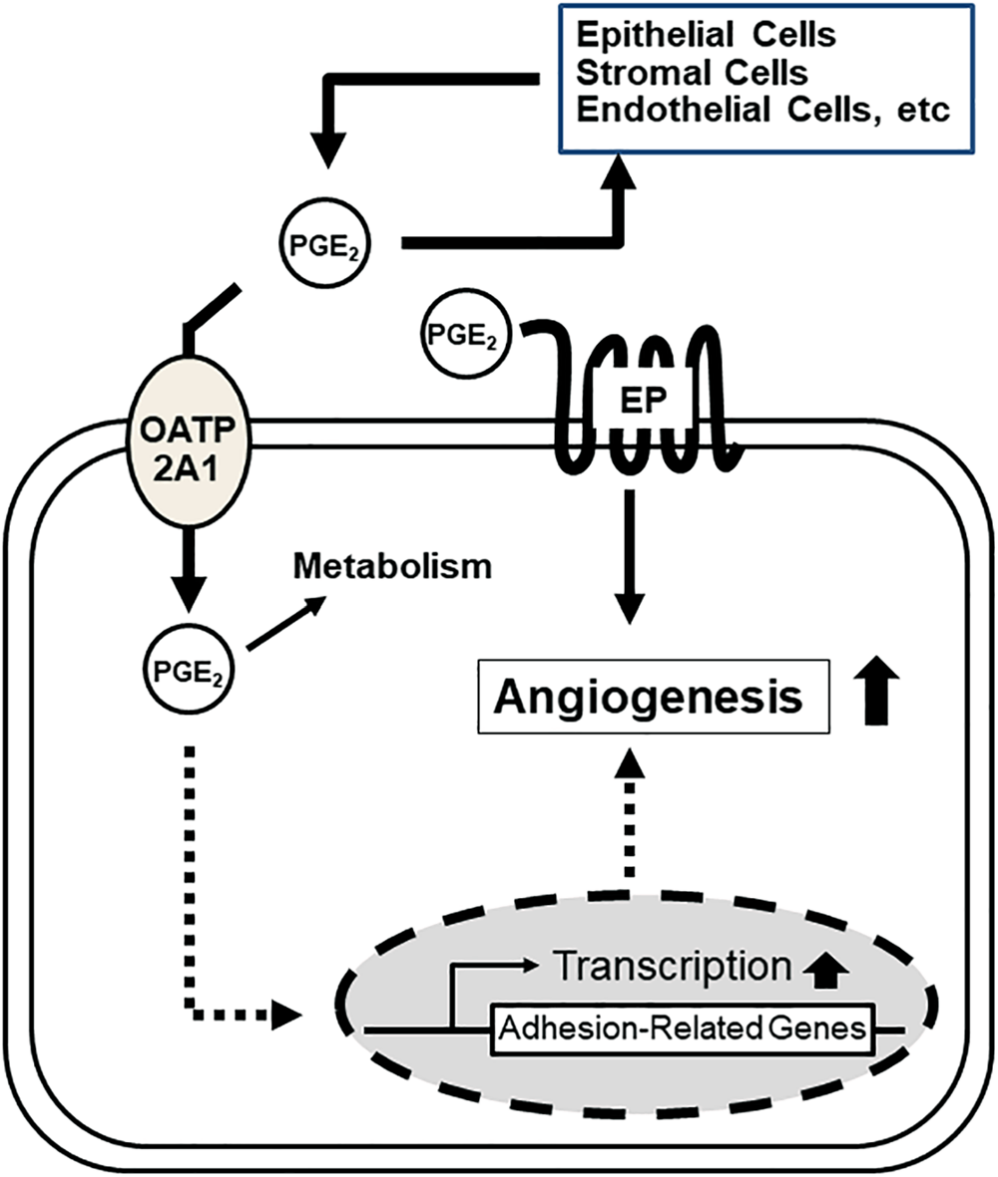
Hypothesized role of OATP2A1 in intracrine PGE_2_ signalling. Secreted PGE_2_ from any cells, including normal and transformed epithelial cells, stromal cells (e.g. fibroblasts and macrophages) and endothelial cells may contribute to PGE_2_ signalling via cell surface EP receptors in endothelial cells, which affects angiogenesis. OATP2A1 expressed in the plasma membranes and/or subapical structures in endothelial cells actively imports PGE_2_. Based on the previous research PGE_2_ taken up by cells is assumed to be metabolized; however, our present study suggests that it may transmit signalling through intracellular intermediates to promote transcription of adhesion molecules.

To elucidate how OATP2A1 affected angiogenesis we used the “tube formation” assay in human vascular endothelial cells. Tube formation in these cells was first evaluated with CM from the LoVo colon cancer cell line. The tube formation was reduced by varying combination of either COX inhibition (indomethacin) or EP antagonists, or BSP (an inhibitor of OATP2A1) suggesting each one of these factors contributes to angiogenesis (Fig. 4a). Our initial experiments did not determine if PGE_2_ alone affected tube formation because OATP2A1 can transport multiple prostaglandins and we did not want to exclude any one prostaglandin from consideration. Subsequently, we demonstrated that PGE_2_ alone affected angiogenesis using the wound healing assay in EBM^TM^ (Fig. 4 and 7). Driving angiogenesis related to OATP2A1-mediated uptake might be attributed to PGE_2_ activating the peroxisome proliferator-activated receptor (PPAR) γ^38,39^, which is likely considering PPARγ is expressed in endothelial cells^40^. PPARγ activation might induce angiogenesis in endothelial cells, possibly by COX-2 upregulation which could increase PGE_2_ production. The reduction of tube formation by the COX-2 inhibitor, indomethacin, in HUVECs is consistent with this proposition. We also demonstrated that some of the HUVEC angiogenesis is mediated by EP receptor activation because the EP receptor antagonists suppressed tube formation (Fig. 4). At this point, we have not elucidated the relative importance of the EP receptor mediated pathway vs the “intracrine” pathway suggested by our findings with the *Slco2a1* knockout mouse and our *in vitro* model systems. A hypothesized model is illustrated in Fig. 8. Indeed, the biological action of intracellular PGE_2_ has been shown in mammalian endothelial cells^41^, human prostate cancer PC3 cells^42^, and human endometrial stromal cells^43^. Hence, blocking both pathways might be synergistically effective to suppress angiogenesis. Future studies will dissect the relative importance of each of these components to angiogenesis and colon cancer tumorogenesis.

The present findings suggest OATP2A1 modulates angiogenesis by promoting neovascularization through PGE_2_ uptake. While a previous study showed that OATP2A1 mRNA was reduced in colon tumours^20^ it did relate this to patient outcome. However, the role of OATP2A1 expression in colon cancer patient survival was to our knowledge unknown. Our preliminary studies, in humans, suggest higher expression of *SLCO2A1* is a poor prognostic factor. However, a caveat to this interpretation is that this data is from a small cohort and does not quite achieve statistical significance (Supplementary Figure S2). Certainly future studies, are necessary to establish a clear relationship between SLCO2A1 expression in colorectal tumours and disease survival. Such studies may pave the way toward a therapeutic approach where OATP2A1 is used as a pharmacological target to suppress colorectal tumour progression. On the other hand, loss-of-function mutations of *SLCO2A1* is a causative gene for primary hypertrophic osteoarthropathy and chronic non-specific ulcers in small intestine, which are related to aberrant catabolism of PGE_2_^35,44,45^; therefore, adverse effects of OATP2A1 blocking may be concerned as well.

In conclusion, in *Slco2a1* ^−/−^ */Apc* ^*Δ716/*+^ mice, the reduced number of large polyps associated with increased survival suggested that absence of *Slco2a1* delays or blocks the formation of large polyps, possibly by suppressing angiogenesis, due to reduced PGE_2_ uptake. This would fit with the increase in small polyps in mice with *Slco2a1* insufficiency. Our mouse model showed that the amount of OATP2A1 impacted colon cancer survival, thereby implying pharmacological blockade of OATP2A1 may increase colon cancer survival. Indeed, OATP2A1 expression in vascular endothelial cells coupled with our *in vitro* studies showing that angiogenesis is suppressed in HUVECs by OATP2A1 inhibition strongly supports this as a potential pharmacological target to improve colon cancer outcomes.

## Materials and Methods

### Materials

[5,6,8,11,12,14,15-^3^H]PGE_2_ ([^3^H]PGE_2_; 163.6 Ci/mmol) was purchased from PerkinElmer Life Science (Boston, MA). Bromosulfophthalein (BSP) was obtained from Sigma-Aldrich (St. Louis, MO) and Tokyo Chemical Industry (Tokyo, Japan), respectively. All other compounds were commercial products of reagent grade. Anti-mouse Oatp2a1 guinea pig and anti-human OATP2A1 rabbit polyclonal antibodies were kind gifts from Prof. Ken-ichi Hosoya (University of Toyama)^46^ and Michel A. Fortier (Université Laval, Canada)^43^, respectively.

### Animals and quantification of intestinal polyps

All animal experimentation was carried out in accordance with the requirements of Kanazawa University Institutional Animal Care and Use Committee, and animal experiment protocols performed in this study were approved by the committee (Approved numbers; AP-153511 and AP-163750). Constructions of *Apc* ^*Δ716/*+^ and *Slco2a1* ^−/−^ mice were described previously^21,47^. *Apc* ^*Δ716/*+^ mice were from a C57BL/6 strain^48^. The null allele of *Slco2a1* was introduced into the *Apc* ^*Δ716/*+^ mice by intercrossing the *Apc* ^*Δ716/*+^ mice with *Slco2a1* ^−/−^ mice to produce mice with a mixed genetic background of BL/6 and SV129. Compound heterozygotes for *Slco2a1* and *Apc* (*Slco2a1* ^+/−^ */Apc* ^*Δ716/*+^) on the mixed genetic background were interbreed to generate *Apc* mutant mice with three different *Slco2a1* genotypes (*Slco2a1* ^+*/*+^ */Apc* ^*Δ716/*+^, *Slco2a1* ^+/−^ */Apc* ^*Δ716/*+^ and *Slco2a1* ^−/−^ */Apc* ^*Δ716/*+*−*^). Polyps in the small and large intestines of these mice were observed at age of 13 weeks (16.9–25.3 g, no significant difference between mice with different genotypes), and their number were counted under a stereo microscope at ×30–60 magnification described as previously^21^. Animal survival was analysed by Kaplan-Meier methods, and then compared by generalized Wilcoxon test.

### Immunohistochemistry

Tissue samples were excised, and then fixed with 4% paraformaldehyde. Briefly, for light-microscopic analysis, paraffin-embedded sections were incubated with guinea pig anti-Slco2a1 IgG (1:100 dilution, overnight at 4 °C), rat anti-CD34 IgG (1:50 dilution, overnight at 4 °C, BD Biosciences, NJ) or anti-F4/80 IgG (1:100 dilution, 2 h at room temperature (rt), AbD Serotec, Raleigh, NC), followed by biotinylated or fluorescence-labelled secondary antibodies (1: 200–400 dilution). For DAB staining, the biotinylated IgG labelled-sections were reacted with horseradish peroxidase-conjugated streptavidin (ThermoFisher Scientific, MA), and developed with 3,3′-diaminobenzidine (Vector Laboratories, Burlingame, CA). The sections were observed with a conventional fluorescence microscope BZ-9000 (Keyence, Osaka, Japan) or a confocal laser microscope LSM710 (Carl Zeiss, Oberkochen, Germany). For evaluation of microvascular density (MVD), CD34-positive area was quantified by using Image J software^49^.

### Tube formation assay

HUVECs (Lonza, Basel, Switzerland) were cultured in endothelial cell basal medium (EBM™, Cat. No. CC-3121, Lonza) supplemented with endothelial cell growth medium (EGM™-2, Cat. No. CC-4176, Lonza) and 2% FBS (Biosera, Kansas City, MO). Matrigel® (Corning, New York, NY) was solidified in a 96-well plate (45 µL/well), and then 9000 HUVECs were placed on each Matrigel® with Ham’s F-12 supplemented with 10% FBS, 100 U/mL Penicillin G and 100 μg/mL streptomycin (Wako Pure Chemical Industries, Osaka, Japan) or conditioned medium (CM) from human cancer LoVo cells, which were cultured at a density of 2 × 10^5^ cells/cm^2^ in 2 mL growth medium for 24 h. After 4 h-incubation at 37 °C, tube formation was evaluated by measuring length of capillary-like structures in two-dimensional microscope images under BZ-9000 using Image J software^49^.

### Wound healing assay

HUVECs were seeded into a multiple well plate at 1.8 × 10^4^ cells/cm^2^ in EBM™ supplemented with EGM™-2 and the assay was conducted as described previously^50^. Next day each confluent monolayer was scraped by a 200-μL plastic pipette tip to make a cell-free area and then detached cells were washed off with EBM™. The cells were incubated at 37 °C in the absence or presence of EP antagonists or an OATP2A1 inhibitor BSP in FBS-free EBM™ supplemented with EGM™-2. To assess effect of exogenous PGE_2_, HUVECs were pre-treated with indomethacin (100 μM) for 16 hrs, and then the assay was performed in the presence or absence of BSP (25 μM) in the EGM^TM^-2 containing indomethacin. The cells were observed at 0 and 10 hrs using BZ-9000 and the distance between the edges of the cell-free areas was measured using Image J software^49^.

### Knockdown of OATP2A1 in HUVECs

OATP2A1 was knocked down as described in previously^48^. Briefly, HUVECs were plated at a density of 0.25 × 10^6^ cells/cm^2^, and then were transfected with 10 nM non-specific (NS) siRNA (Silencer® Select Negative Control #1 siRNA) or a mixture of two siRNAs to *SLCO2A1* gene (Silencer® Select Validated siRNA s13097 and s13098), using Lipofectamine RNAi Max® (Life Technology) according to the manufacturer’s protocol. After the cells were cultured for 48 hrs, mRNA expression was measured by quantitative RT-PCR using oligonucleotide primers specific to *SLCO2A1* (sense; 5′-ctgtggagacaat ggaatcgag-3′, antisense; 5′-cacgatcctgtctttgctgaag-3′), and then normalized with that of HPRT as previously described^51^. Protein expression of OATP2A1 was detected by immunocytochemistry as described below.

### Immunocytochemistry for HUVECs

OATP2A1 was immunostained as described previously^48^. HUVECs were plated on glass slides (BD Falcon, Franklin Lakes, NJ) at a density of 5 × 10^4^ cells/0.7 cm^2^. The cells were fixed in 4% paraformaldehyde, and permeabilized with 0.01% (w/v) Triton X100 in PBS. Immunoreaction was performed by incubating the cells with a 1:200 dilution of anti-human OATP2A1 rabbit polyclonal antibody for 3 hrs at rt, followed by staining with a 1:400 dilution of AlexaFluor® 594-conjugated anti-rabbit IgG (Life Technologies) for 1 hr at rt. The cells were counterstained with Hoechst 33342 (2 μg/mL) for nucleus (blue), and then mounted with Vectashield® (Vector Laboratories, Peterborough, UK). Fluorescence was examined by the use of a confocal laser microscope (LSM710, Carl Zeiss, Göttingen, Germany).

### PGE_2_ uptake by HUVECs

Cells were cultured on collagen-coated plate at a density of 0.5 × 10^5^ cells/cm^2^ for 2 days, and then used for [^3^H]PGE_2_ uptake was undertaken in the absence or presence of an OATP inhibitor as described before^52^. Intracellular accumulation of [^3^H]PGE_2_ was evaluated by measuring radioactivity in the cell lysates using a liquid scintillation counter (Hitachi Aloka Medical, Tokyo, Japan), and shown as cell-to-medium ratio normalized by protein content (μL/mg protein).

### *In vivo* angiogenesis

*In vivo* angiogenesis was evaluated based on the previous studies^28,53^. Sponges (12 mm dia. × 3 mm height) were surgically implanted subcutaneously onto the dorsum of mice with *Slco2a1* wildtype (*Slco2a1* ^+/+^) or null (*Slco2a1* ^−/−^) alleles, both of which have mixed genetic background of BL/6 and SV129 (ages 11–30 weeks) under general anaesthesia with pentobarbital sodium (50 mg/kg, i.p.). *Slco2a1* ^+/+^ mice were randomly divided into two groups, and sterilized physiological saline (100 μL, vehicle) or vehicle containing 5 nmol of an OATP inhibitor BSP was injected for 9 consecutive days into the implanted sponge, starting the day after the surgery, and then all animals were monitored daily and sacrificed at the 10^th^ post-surgery day. The sponges were carefully removed and the contents were homogenized in sterilized water and centrifuged at 5000 × g for 10 min. Haemoglobin contained in the supernatant was quantified with Haemoglobin B-Test Wako according to manufacturer’s protocol (Wako Pure Chemical Industries).

### Statistical analysis

Student’s t-test was used to assess significance of difference between *in vitro* assay results, with p < 0.05 as a criterion of significance.

### Data availability

All data generated or analysed during this study are included in this published article (and its Supplementary Information files).

## Electronic supplementary material

Supplementary Figures
