# Supplementary material for: A novel role for OATP2A1/SLCO2A1 in a murine model of colon cancer
Source: Sci Rep. 2017 Nov 29;7:16567. doi: 10.1038/s41598-017-16738-y (PMC5707394; doi:10.1038/s41598-017-16738-y)
Supplement: Supplementary file 1 — Supplementary Figures [file 41598_2017_16738_MOESM1_ESM.pdf]

A novel role for OATP2A1/*SLCO2A1* in a murine model of colon cancer

Takeo Nakanishi[1*], Yasuhiro Ohno[1], Rika Aotani[1], Shio Maruyama[1], Hiroaki Shimada[1,4], Shunsuke Kamo[1], Hiroko Oshima[2], Masanobu Oshima[2], John D. Schuetz[3], Ikumi Tamai[1]

Affiliation

[1]Department of Membrane Transport and Biopharmaceutics, Faculty of Pharmaceutical Science

[2]Cancer Research Institute, Kanazawa University, Kanazawa, Japan

[3]Department of Pharmaceutical Sciences, St. Jude Children's Research Hospital, Memphis, TN, USA

[4]Currnet address; Faculty of Pharmacy, Kindai University, Higashiosaka, Osaka, Japan

Corresponding Author

Takeo Nakanishi

Kakuma-machi, Kanazawa 920-1192, Japan

Department of Membrane Transport and Biopharmaceutics, Faculty of Pharmaceutical Sciences, Kakuma-machi, Kanazawa, Ishikawa, 920-1192, Japan,

Tel: +81-76-234-4421, Fax: +81-76-264-6284,

E-mail: nakanish@p.kanazawa-u.ac.jp

Supplementary Figures

Supplementary Figure S1

EP4 expression in polyps in the small intestine

Method

Tissue samples were excised, and then fixed with 4 % paraformaldehyde. Briefly, for light-microscopic analysis, paraffin-embedded sections were incubated with rabbit anti-mouse EP4 IgG (1:10 dilution, overnight at 4°C, Cayman Chemical, Ann Arbor, MI), and followed by horseradish peroxidase-conjugated secondary antibodies (1: 100-200 dilution). DAB stain was developed with 3,3'-diaminobenzidine (Nacalai Tesque, Kyoto, Japan). The sections were observed with a light microscopy (Nikon E200, Tokyo, Japan)

Result

EP4 expression was determined by means immunohistochemistry in polyps of the small intestines from *Slco2a1*$^{+/+}$ and *Slco2a1*$^{-/-}$/*Apc*$^{\Delta 716/+}$. The picture shown as Supplementary Figure S1 represents the at least three individual animals in each group. DAB stain was clearly developed in the epithelial cells and some stromal cell, and blood vessels. Apparently significant changes were unlikely observed between *Slco2a1*$^{+/+}$ and *Slco2a1*$^{-/-}$/*Apc*$^{\Delta 716/+}$ mice.

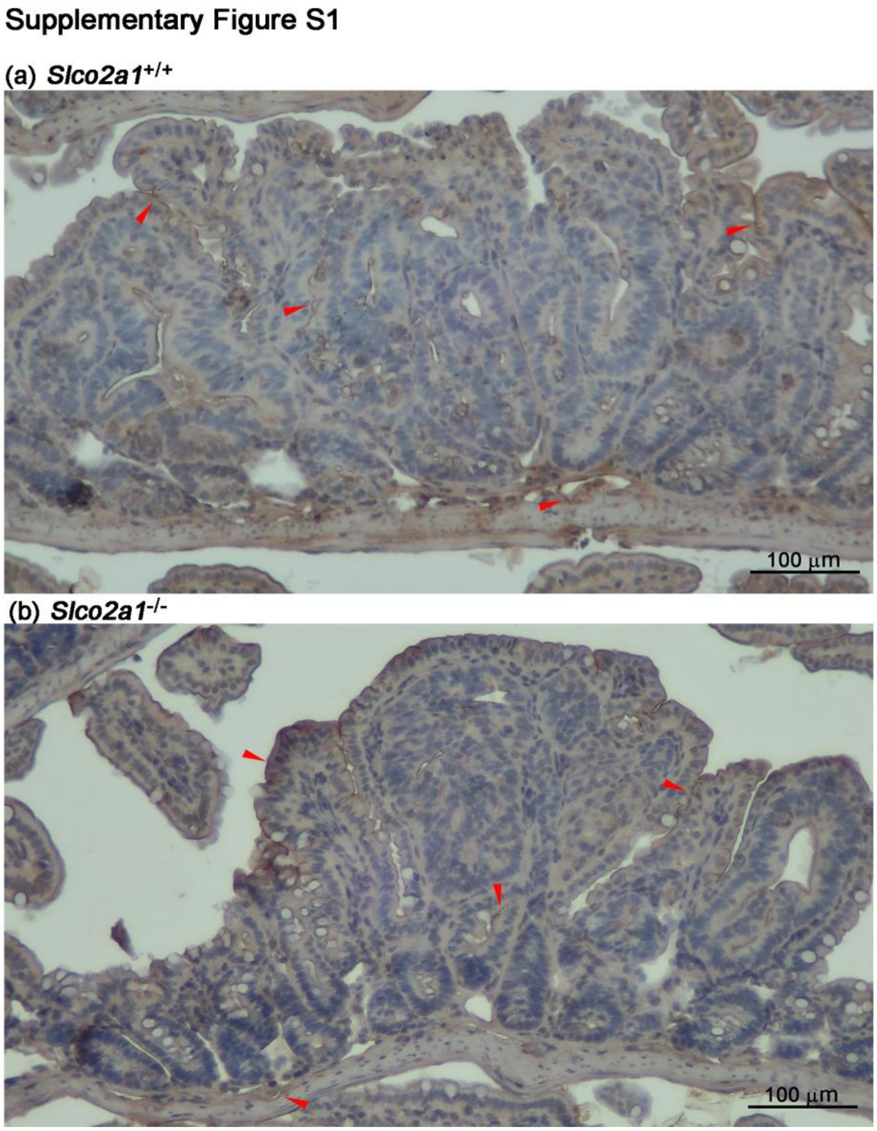

Supplementary Figure S2

Analysis of SLCO2A1 expression in human colon cancer patients

To assess any association of *SLCO2A1* expression with colorectal cancer survival, the Oncomine gene expression database was interrogated for mRNA levels of *SLCO2A1* related to colon cancer patient outcome using datasets from published studies (1). The one year survival data from the Cancer Gene Atlas (TCGA) was analysed by an unpaired t-test using Welch's correction. The survival data was analysed by the Mantel-Cox log-rank test.

Result

Using publically available Oncomine database (1), we compared patients with colon adenocarcinoma where tumour *SLCO2A1* mRNA expression and survival data were available (**Supplementary Fig. S2**). Only 25 cases with one year survival data (**Supplementary Fig. S2a**) and 20 cases with overall survival data (**Supplementary Fig. S2b**) were found that also had tumour *SLCO2A1* mRNA expression data. Although not reaching statistical significance, an association was noted between higher expression of *SLCO2A1* mRNA in tumour tissue and shortened survival (p = 0.14, **Supplementary Fig. S2b**). These observations led us to test the hypothesis that *SLCO2A1* expression impacts colon cancer disease progression in a mouse colon cancer model.

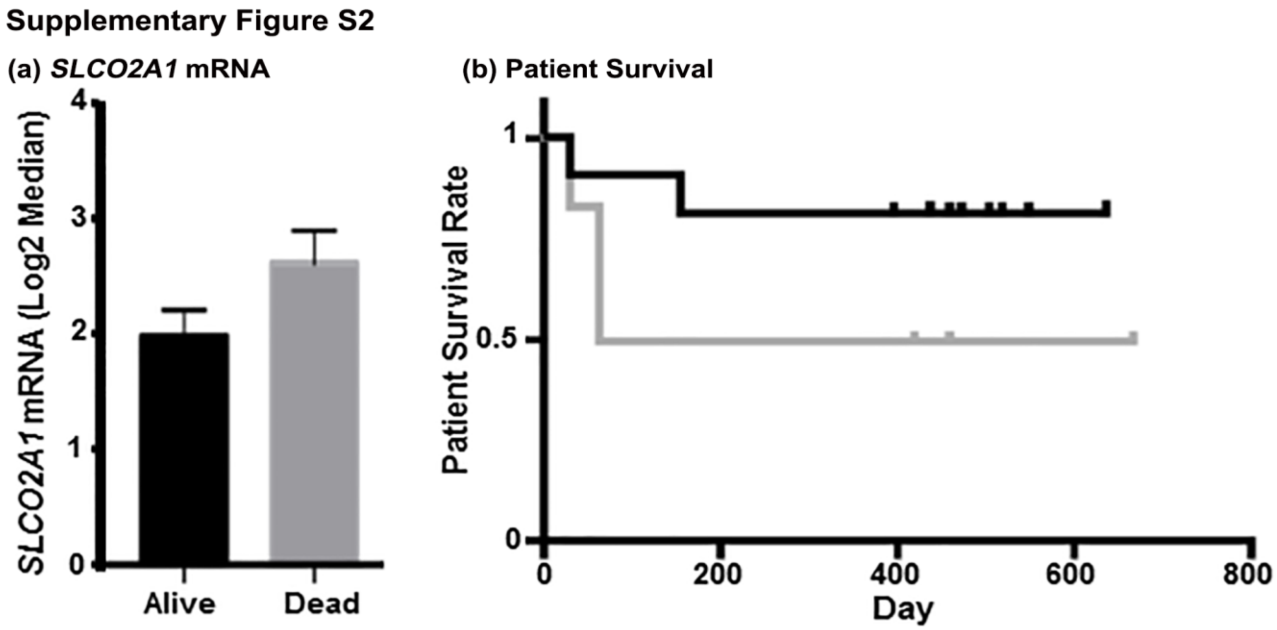

Legend for Supplementary Figure S2

***SLCO2A1* expression affects colon adenocarcinoma patients**

(a) *SLCO2A1* mRNA expression in colon adenocarcinoma patients who survived (black, n = 17) and died (grey, n = 8) one year according to the Cancer Genome Atlas (p = 0.1). Data was obtained from Oncomine data base (1). (b) Survival curves of colon adenocarcinoma patients with higher (grey, n = 9) and lower (solid, n = 11) *SLCO2A1* mRNA expression than median value (p = 0.14).

Reference

1. Rhodes DR, Kalyana-Sundaram S, Mahavisno V, Varambally R, Yu J, Briggs BB, et al. Oncomine 3.0: genes, pathways, and networks in a collection of 18,000 cancer gene expression profiles. *Neoplasia* **2007**;9:166-180.
